# Dynamic annealing in Ge studied by pulsed ion beams

**DOI:** 10.1038/s41598-017-13161-1

**Published:** 2017-10-13

**Authors:** J. B. Wallace, L. B. Bayu Aji, L. Shao, S. O. Kucheyev

**Affiliations:** 1 Lawrence Livermore National Laboratory, Livermore, California 94550 USA; 2 Department of Nuclear Engineering, Texas A&M University, College Station, Texas 77843 USA

## Abstract

The formation of radiation damage in Ge above room temperature is dominated by complex dynamic annealing processes, involving migration and interaction of ballistically-generated point defects. Here, we study the dynamics of radiation defects in Ge in the temperature range of 100–160 °C under pulsed beam irradiation with 500 keV Ar ions when the total ion fluence is split into a train of equal square pulses. By varying the passive portion of the beam duty cycle, we measure a characteristic time constant of dynamic annealing, which rapidly decreases from ~8 to 0.3 ms with increasing temperature. By varying the active portion of the beam duty cycle, we measure an effective diffusion length of ~38 nm at 110 °C. Results reveal a major change in the dominant dynamic annealing process at a critical transition temperature of ~130 °C. The two dominant dynamic annealing processes have an order of magnitude different activation energies of 0.13 and 1.3 eV.

## Introduction

For the past decade, there has been renewed interest in the use of Ge for high-speed, low-power electronics^1^. Ion implantation is the preferred tool for selective-area doping of semiconductor devices. However, implantation of dopants is accompanied by the formation of lattice defects that strongly and often deleteriously influence material properties. Hence, numerous recent studies have focused on ion implantation damage in Ge^2–7^.

It has been well established that the buildup of radiation damage in Ge, particularly above room temperature, is complicated by pronounced dynamic annealing (DA) processes^2–13^. These involve migration, recombination, and clustering of mobile point defects *during irradiation*. Such DA is commonly manifested as a dependence of stable lattice disorder on the dose rate and sample temperature (*T*). Despite previous efforts^2–13^, the current understanding of DA in Ge is very limited, and some very basic questions about defect interaction remain unanswered. For example, after the thermalization of ballistic collision cascades, how long do mobile point defects survive and how far do they diffuse while participating in DA processes? Previous estimates of the defect relaxation time constant (*τ*) in Ge cover an astonishingly wide range of 12 orders of magnitude (10^−11^–10^1^ s)^13,14^. We are unaware of any previous measurements of the effective diffusion length of mobile defects (*L*_*d*_) in ion-bombarded Ge.

Here, we use a recently developed pulsed ion beam method^15–21^ to study the dynamics of radiation defect interaction in Ge bombarded with 500 keV Ar ions. We measure *τ* values of ~10^−4^–10^−2^ s (monotonically decreasing with *T*) and an *L*_*d*_ of ~38 nm at 110 °C. Furthermore, the *τ*(*T*) dependence reveals a change in the dominant DA process at ~130 °C, which is accompanied by an order of magnitude change in the activation energy of the dominant DA process.

## Experimental

Czochralski grown (100) Ge single crystals doped with Ga (with a resistivity of ~0.03 Ω cm) were bombarded in the *T* range of 100–160 °C with 500 keV ^40^Ar^+^ ions at 7° off the [100] direction. Previous studies^8–11^ have found pronounced DA in this *T* range. To improve thermal contact, the samples were attached to a Cu sample holder with Ag paste. All irradiations were performed in a broad beam mode^15^. Ion beam pulsing was achieved by applying high voltage pulses to a pair of parallel plates deflecting the beam off the final beam defining aperture. The 4 MV ion accelerator (National Electrostatics Corporation, model 4UH) at Lawrence Livermore National Laboratory was used for both ion irradiation and ion beam analysis.

**Figure 1:**
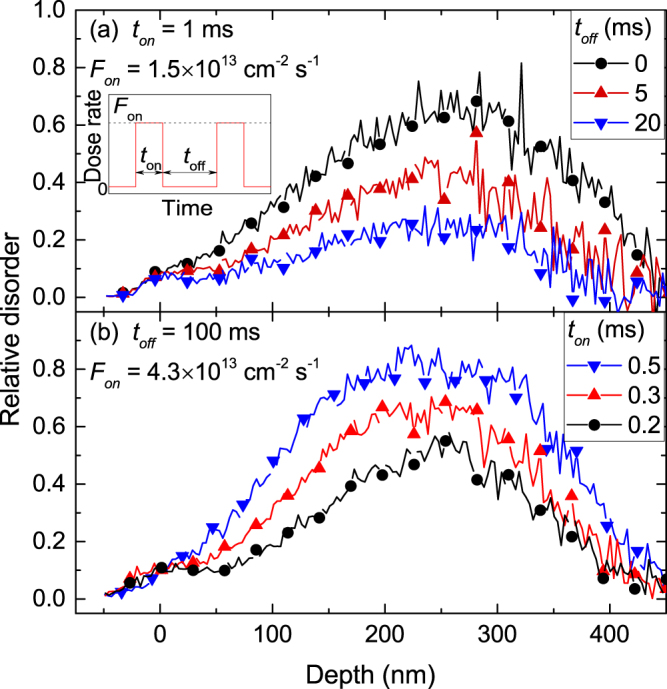
Selected depth profiles of relative disorder in Ge bombarded at 110 °C with a pulsed beam of 500 keV Ar ions with *F*_*on*_, *t*_*on*_, and *t*_*off*_ given in the legends at total fluences of (**a**) 4.9 × 10^13^ cm^−2^ and (**b**) 7.5 × 10^13^ cm^−2^. For clarity, only every 10th experimental point is depicted. Panel (a) is a pulsed beam measurement of *τ*, whereas (**b**) is a measurement of *L*_*d*_. The inset in (**a**) is a schematic of the time dependence of the instantaneous dose rate for pulsed beam irradiation, defining *t*_*on*_, *t*_*off*_, and *F*_*on*_.

Similar to our previous work^15–19^, for *τ* measurements, the total ion fluence was split into a train of equal square pulses, each with an instantaneous dose rate (*F*_*on*_) of ~1.5×10^13^ cm^−2^ s^−1^ and duration (*t*_*on*_) of 1 ms. Adjacent pulses in such *τ* measurements were separated by time *t*_*off*_, which was varied between 0.2 and 50 ms. For *L*_*d*_ measurements^17,20,21^, the total fluence was delivered as a train of pulses with duration (*t*_*on*_) varying between 0.2 and 1 ms, each with an instantaneous dose rate (*F*_*on*_) of ~4.3×10^13^ cm^−2^ s^−1^, separated by a *t*_*off*_ of 100 ms, which, as will be shown below, is much greater than the *τ* values. The inset in Fig. 1(a) shows a schematic of the time dependence of the instantaneous dose rate and defines the pulsing parameters (*t*_*on*_, *t*_*off*_, and *F*_*on*_). A more detailed description of the experimental arrangement can be found elsewhere^15–19^.

The dependence of stable lattice damage on *t*_*off*_ and *t*_*on*_ was studied *ex-situ* at room *T* by ion channeling. Depth profiles of lattice disorder were measured with 2 MeV ^4^He^+^ ions incident along the [100] direction and backscattered into a detector at 164° relative to the incident beam direction. Spectra were analyzed with one of the conventional algorithms^22^ for extracting the effective number of scattering centers (referred to below as “relative disorder”). Values of average bulk disorder (*n*), discussed below, were obtained by averaging depth profiles of relative disorder over 15 channels (corresponding to an ~60-nm-wide region) centered on the maximum of the bulk damage peak. Error bars of *n* are standard deviations. Ion fluences in *τ* measurements at different *T*s were chosen such that, for continuous beam irradiation, *n* was in the range of 0.5–0.8 (with *n* = 1 corresponding to full amorphization). The nuclear energy loss profile was calculated with the TRIM code (version SRIM-2013.00)^23^ with an atomic concentration of Ge of 4.4 × 10^22^ atoms cm^−3^ and a threshold energy for atomic displacements of 15 eV.

## Results and Discussion

Figure 1(a) shows representative depth profiles of relative disorder for bombardment at *T* = 110 °C with continuous (*t*_*off*_ = 0 ms) and pulsed (*t*_*off*_ = 5 and 20 ms) beams for *τ* measurements. Figure 1(b) shows corresponding profiles for bombardment at *T* = 110 °C with pulsed (*t*_*on*_ = 0.2, 0.3, and 0.5 ms) beams for *L*_*d*_ measurements. The depth profiles for all irradiations herein, at *T*s of 100–160 °C, have qualitatively similar shapes. They exhibit a major peak in the crystal bulk centered on ~270 nm, which corresponds to the maximum of the nuclear energy loss profile for 500 keV Ar ions. These observations are consistent with previous studies of radiation damage in Ge at room *T* ^3,6,7^.

**Figure 2:**
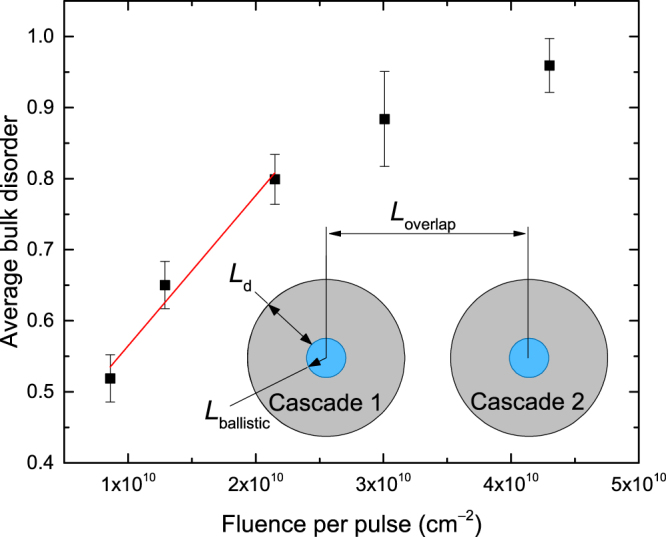
Relative average bulk disorder (*n*) in Ge samples bombarded at 110 °C with a pulsed beam of 500 keV Ar ions with *F*_*on*_ = 4.3 × 10^13^ cm^−2^ s^−1^ and *t*_*off*_ = 100 ms to the same total fluence of 1.5 × 10^14^ cm^−2^ as a function of the fluence per pulse (*F*_*on*_ *t*_*on*_). Linear fitting, shown by the solid line, gives an effective diffusion length (*L*_*d*_) of 38 ± 6 nm. The schematic in the inset shows two adjacent collision cascades created in the same pulse, illustrating the parameters *L*_*overlap*_, *L*_*d*_, and *L*_*ballistic*_ described in the text.

Figure 1(b) shows that *n* increases with increasing *t*_*on*_ when all the other irradiation parameters are kept constant. These results are more clearly shown in Fig. 2, where *n* is plotted as a function of fluence per pulse (*F*_*on*_ *t*_*on*_). As discussed previously^17,20,21^, in such measurements of *n*(*F*_*on*_ *t*_*on*_) dependencies with ${t}_{off}\gg \tau$, the interaction between mobile defects generated in different pulses is suppressed, and the *n*(*F*_*on*_ *t*_*on*_) dependence reflects the interaction of mobile defects created in different cascades within the same pulse. Such inter-cascade defect interaction processes become pronounced when the average lateral distance between the centers of adjacent collision cascades in each pulse $({L}_{overlap}\approx \tfrac{1}{\sqrt{{F}_{on}{t}_{on}}})$ is comparable to or smaller than the effective diffusion length of mobile defects: ${L}_{overlap}\mathop{ < }\limits_{ \tilde {}}2{L}_{d}$ (see the inset in Fig. 2)^17,20,21^. For relatively heavy ion bombardment such as used in the present study, the average lateral dimensions of ballistic sub-cascades, *L*_*ballistic*_, also shown in the inset in Fig. 2, are ~1–2 nm, which is much smaller than *L*_*d*_ and, hence, can be omitted in these estimates^17,20,21^.

For broad beam bombardment with statistically uncorrelated ion trajectories of the present study, ion impacts obey Poisson statistics with a Poisson coefficient of $4{L}_{d}^{2}{F}_{on}{t}_{on}$ ^21^, where $4{L}_{d}^{2}$ is the effective area of a cascade after defect out-diffusion. For *t*_*on*_ < *τ* (i.e., when defect relaxation processes during the active part of each pulse, *t*_*on*_, can be neglected), the average density of elementary mobile point defects within collision cascades after each pulse can be approximated by an average density of atomic displacements: ${\rho }_{displacements}=g(\tfrac{1}{4{L}_{d}^{2}}+{F}_{on}{t}_{on})$, where *g* is the the number of atomic displacements produced by an ion per unit of depth. If the efficiency of stable damage formation scales linearly with the density of mobile defects (which is a good approximation for low *t*_*on*_ values), $n\propto {\rho }_{displacements}\propto 1+4{L}_{d}^{2}{F}_{on}{t}_{on}$. This equation is used to fit the linear portion of *n*(*t*_*on*_) at low *t*_*on*_ values (the solid line in Fig. 2), revealing an *L*_*d*_ of ~38 ± 6 nm. This *L*_*d*_ is larger than the value of ~10 nm in Ar-ion-bombarded 3*C*-SiC reported recently^21^. Interestingly, an *L*_*d*_ of ~38 nm is very similar to that found for Si under 500 keV Ar ion irradiation at room *T* ^17^.

**Figure 3:**
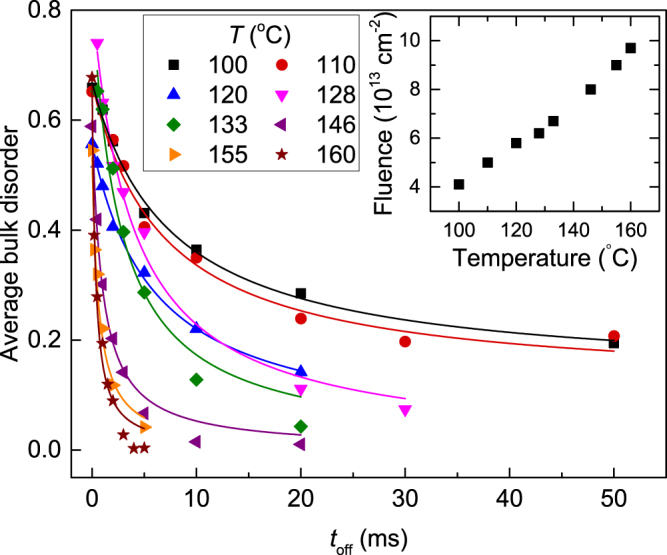
Relative average bulk disorder (*n*) in Ge bombarded with a pulsed beam of 500 keV Ar ions with *F*_*on*_ = 1.5 × 10^13^ cm^−2^ s^−1^ and *t*_*on*_ = 1 ms as a function of the passive portion of the beam duty cycle (*t*_*off*_) at different *T*s given in the legend. Fitting curves with the second order decay equation are shown by solid lines. The inset shows the temperature dependence of the total ion fluence required to achieve a relative bulk disorder level of ~0.5–0.8 for continuous beam irradiation.

Figure 1(a) also shows that *n* decreases with increasing *t*_*off*_. Such an experimental *n*(*t*_*off*_) dependence is used to evaluate *τ*. This is better illustrated in Fig. 3, which summarizes *n*(*t*_*off*_) dependencies for all the *T*s studied, at total fluences shown in the inset. It is seen from Fig. 3 that, for all the cases, *n* monotonically decreases with increasing *t*_*off*_. Solid lines in Fig. 3 are fits of *n*(*t*_*off*_) dependencies via the Marquardt-Levenberg algorithm^24^ with a second order decay equation $(n({t}_{off})={n}_{\infty }+\tfrac{n\mathrm{(0)}-{n}_{\infty }}{1+\tfrac{{t}_{off}}{{\tau }_{2}}})$. Here, *τ*_2_ is the characteristic decay time constant measured by fitting to the second order decay equation, and *n*_∞_ is relative disorder for ${t}_{off}\gg {\tau }_{2}$.

We find that, across the *T* range studied, the best fits to *n*(*t*_*off*_) dependencies of Fig. 3 alternate between the two simplest decay process equations: the first order (*n*(*t*_*off*_) = *n*_∞_ + (*n*(0) − *n*_∞_) exp (−*t*_*off*_/*τ*_1_)) and second order decay equations. This finding is in contrast to results of our recent pulsed beam study^19^ of Si that has revealed a clear switch from the second to the first order decay at a certain *T*. However, both first and second order fits for Ge shown in Fig. 3 have *R*-squared values of >0.9 (i.e., coefficients of determination, which are commonly used as a measure of the goodness of fit)^25^. An example of the first order decay process is the trapping of interstitials or vacancies at sinks, while vacancy–interstitial annihilation and the formation of di-vacancies are examples of the second order kinetic processes. Although it is tempting to associate the best fitting decay curves with some specific defect interaction processes, such assertions will require future modeling work as, for example, in a recent study of ion-bombarded Si^19^.

**Figure 4:**
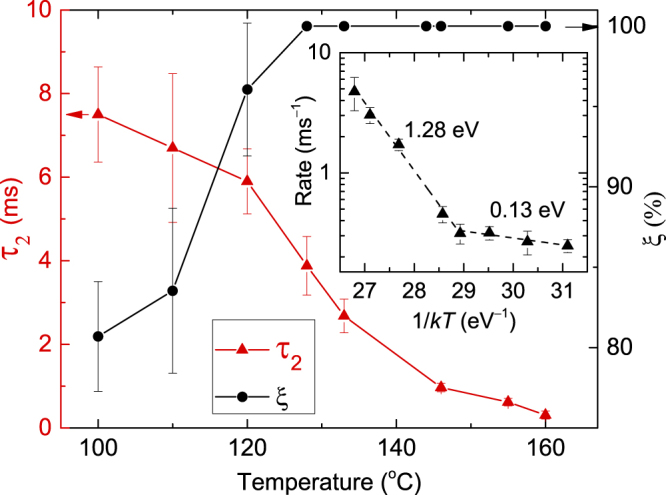
Temperature dependencies of the effective time constant of dynamic annealing (*τ*_2_, left axis) and the dynamic annealing efficiency (*ξ*, right axis) for Ge bombarded with 500 keV Ar ions. The inset shows an Arrhenius plot of the dynamic annealing rate. Dashed lines in the inset are results of linear fitting, revealing activation energies of 1.28 ± 0.07 eV and 0.13 ± 0.03 eV, above and below 130 °C, respectively.

The *τ*_2_(*T*) dependence is plotted in Fig. 4 (left axis), revealing a monotonic decrease with increasing *T*. Figure 4 (right axis) shows the *T* dependence of the DA efficiency (*ξ*), which we define as follows^15,17^: *ξ* = (*n*(0) − *n*_∞_)/*n*(0). For our choice of the pulsing parameters, *ξ* is the magnitude of the dose rate effect, reflecting the fraction of mobile defects that participate in DA processes during continuous beam irradiation with a dose rate of *F*_*on*_^17^. Fig. 4 shows a monotonically increasing *ξ*(*T*) dependence up to ~130 °C and saturation at *ξ* ≈ 100% for higher *T*s. This indicates a very strong dose rate effect at $T\mathop{ > }\limits_{ \tilde {}}130\,^\circ {\rm{C}}$, when stable lattice damage forms predominantly in inter-cascade DA processes. In this irradiation regime, the control of the beam shape and both instantaneous and average dose rates becomes critically important since relatively small changes in the dose rate can dramatically affect the level of stable damage, from barely detectable by ion channeling to full lattice amorphization.

As discussed in detail in ref.^17^, in measurements of *τ* based on *n*(*t*_*off*_) dependencies [Figs 1(a) and 3], the fluence per pulse (*F*_*on*_ *t*_*on*_) can be chosen in order to minimize intra-pulse defect interaction, while maximizing the inter-pulse interaction. This occurs when, on average, only one ion impacts onto *L*_*d*_-defined areas during each pulse. This condition is satisfied when ${t}_{on}={t}_{on}^{{L}_{d}}=\tfrac{1}{4{L}_{d}^{2}{F}_{on}}$ ^17^. For *F*_*on*_ = 1.5 × 10^13^ cm^−2^ s^−1^ and *L*_*d*_ = 38 nm, ${t}_{on}^{{L}_{d}}\approx 1.2\,{\rm{ms}}$. Hence, we have selected *t*_*on*_ = 1 ms for all the *τ* measurements of this study.

**Figure 5:**
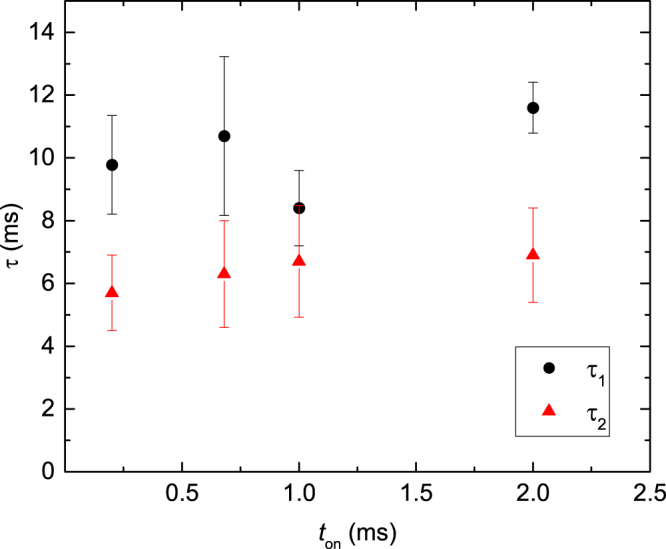
Effective time constants of dynamic annealing (*τ*_1_ and *τ*_2_) for Ge bombarded at 110 °C with 500 keV Ar ions as a function of the active portion of the beam duty cycle (*t*_*on*_) when the fluence per pulsed is kept constant at 2 × 10^10^ cm^−2^ by selecting different instantaneous dose rates (*F*_*on*_). Values of *τ* obtained by fitting experimental *n*(*t*_*off*_) dependencies with either the first and second order decay equation are shown, as indicated in the legend.

As also discussed in detail in ref.^17^, for ${t}_{on} < {t}_{on}^{{L}_{d}}$, the effective time between ion impacts onto *L*_*d*_-defined areas is not the passive part of the beam duty cycle (*t*_*off*_) but ${t}_{off}^{effective}=({t}_{on}+{t}_{off})\,\tfrac{{t}_{on}^{{L}_{d}}}{{t}_{on}}$. So, the defect relaxation time constant *τ* can be more accurately evaluated by the analysis of $n({t}_{off}^{effective})$ dependencies. However, the analysis of data from Fig. 3 as $n({t}_{off}^{effective})$ gives *τ* values that differ only by ~10% from the *τ* values obtained by the analysis of *n*(*t*_*off*_) dependencies and shown in Fig. 4. We have also found that *τ* is independent of the choice of *t*_*on*_ (varied between 0.2 and 2.0 ms) when *F*_*on*_ *t*_*on*_ is kept constant (Fig. 5). All these observations are consistent with a recent detailed discussion of the choice of pulsing parameters^17^.

Figure 4 shows that the *τ* values are in the range of ~10^−4^–10^−2^ s. These are much larger than defect relaxation time scales previously studied by molecular dynamics simulations^14^, which are appropriate for evaluating defect dynamics on time scales $\mathop{ < }\limits_{ \tilde {}}{10}^{-9}\,{\rm{s}}$. The only experimental estimate of *τ* for Ge that we are aware of is by Posselt *et al*.^13^ for focused ion beam (channeled 30 keV Ga) irradiation. They^13^ estimated a lower limit of 10 s at 20 °C and an upper limit of 10 ms at 250 °C. Extrapolating our measurements, we find *τ* values of ~0.5 *μ*s and 40 ms at 250 and 20 °C, respectively. This is consistent with the upper limit (*τ* < 10 ms) but is well below the lower limit (*τ* > 10 s) given by Posselt *et al*.^13^. This apparent inconsistency could be related to a more complex *τ*(*T*) dependence at lower *T*s, different irradiation conditions, or limitations of estimating *τ* based on the dose rate effect^17^.

Comparing DA in Ge to that in other semiconductors, we note that our *τ* values of ~0.3–8 ms in Ge (at 100–160 °C) are similar to those for Si (~0.2–14 ms in a wider *T* range from −20 to 140 °C), 3*C*-SiC (*τ* = 3 ms at 100 °C), and 4*H*-SiC (*τ* of ~1–5 ms at 25–250 °C) recently measured with the pulsed ion beam technique^15–19^. Despite such similarity of the range of the *τ* values measured, the details of defect interaction dynamics are strongly material dependent. First, we note that these previous pulsed-beam studies of different materials^16,18,19^ were performed at different *T*s. Only at 100 °C, do data sets for these four materials overlap, yielding *τ*_1_ values of 8.9 ± 1.5, 0.98 ± 0.07, 4.6 ± 0.8, and 6.9 ± 1.2 ms for Ge, Si, 3*C*-SiC, and 4*H*-SiC, respectively, irradiated with 500 keV Ar ions^16,18,19^. Hence, among these four materials, Ge exhibits the slowest defect interaction dynamics at 100 °C (an order of magnitude slower than for Si). The ratio of *τ* for different materials, however, strongly depends on *T* since different materials have uniquely different *τ*(*T*) dependencies, and more work is currently needed to understand any possible correlation between basic materials properties and *τ* values.

The *τ* parameter describes the dynamic interaction of mobile point defects produced in different pulses and, hence, in different collision cascades. In other words, *τ* is a parameter of inter-cascade (rather than intra-cascade) defect interaction. To gain insight into such inter-cascade defect dynamics, we replot the *τ*_2_(*T*) dependence in Arrhenius coordinates as shown in the inset of Fig. 4. The second order DA rate is defined as $\tfrac{1}{{\tau }_{2}(n\mathrm{(0)}-{n}_{\infty })}$, and *kT* has the usual meaning. Two well defined Arrhenius regions, above and below 130 °C, are clearly revealed in the inset of Fig. 4. Linear fitting of the data gives activation energies of 1.28 ± 0.07 eV and 0.13 ± 0.03 eV, above and below 130 °C, respectively. When the *n*(*t*_*off*_) dependencies from Fig. 3 are fitted with the first order decay equation (and the DA rate defined as $\tfrac{1}{{\tau }_{1}}$), we find activation energies of 1.1 and 0.2 eV, above and below 130 °C, respectively. Hence, the choice of any particular form of the decay equation to fit experimental *n*(*t*_*off*_) dependencies in order to quantify the DA rate is not a critical factor in measurements of activation energies.

How are these activation energies related to energy barriers of basic defect migration and interaction processes? It is tempting to associate the activation energies measured here with migration energies of interstitials and vacancies. This is, however, not straightforward. Indeed, previous estimates of point defect migration energies in Ge are limited and, more importantly, vary widely. All the previous experimental estimates of vacancy and interstitial migration energies in Ge have been indirect, with values ranging from 0.2 to 1.3 eV for vacancies^26–28^ and 0.16 eV for interstitials^27^. Similarly, theoretical studies predict migration energies of 0.1–0.7 eV for vacancies^29^ and 0.3–1.4 eV for interstitials^29,30^. Hence, the identification of the energetic barriers of different defect migration or interaction processes will need to await further studies that could enable future modeling of DA in pulsed-ion-irradiated Ge, similar to what was recently reported for Si^19^, for which elementary defect migration processes are much better understood than for Ge.

Finally, we discuss a transition temperature (*T*_*c*_) of ~130 °C, which is consistent with *T*s previously reported (~114–155 °C)^8–10^ above which damage buildup depends strongly on *T* and the dose rate. It also agrees with the *T* of a post-irradiation defect annealing stage (rather than DA)^31–34^. This *T*_*c*_ is much larger than the *T*s at which primary defects in Ge become mobile, which appears to occur below ~200 K^31,34–36^. Our results clearly show that a *T*_*c*_ of 130 °C corresponds to a change in the dominant DA mechanism. What are these two dominant mechanisms? The fact that *ξ* reaches a saturation of ~100% above *T*_*c*_ could suggest that the defect clusters produced during cascade thermalization and intra-cascade DA are no longer stable, and DA becomes dominated by inter-cascade processes. However, comprehensive theoretical studies, benchmarked against our experimental data, are required to better understand the atomistics of DA in Ge.

## Conclusion

In summary, we have used the pulsed ion beam method to study defect interaction dynamics in Ge bombarded in the *T* range of 100–160 °C with 500 keV Ar ions. Results have revealed that, with increasing *T*, *τ* decreases monotonically in the range of ~0.3–8 ms. We have estimated an *L*_*d*_ of ~38 nm. There is a major change in the dominant DA process at a critical temperature of 130 °C, which separates two defect accumulation regimes characterized by defect relaxation rates with very different activation energies of 0.13 ± 0.03 and 1.28 ± 0.07 eV. These results provide an important step toward the understanding of the mechanisms of radiation damage buildup in Ge.
